# Adjuvant NY-ESO-1 vaccine immunotherapy in high-risk resected melanoma: a retrospective cohort analysis

**DOI:** 10.1186/s40425-018-0345-7

**Published:** 2018-05-18

**Authors:** Michael Lattanzi, Joseph Han, Una Moran, Kierstin Utter, Jeremy Tchack, Rachel Lubong Sabado, Russell Berman, Richard Shapiro, Hsin-Hui Huang, Iman Osman, Nina Bhardwaj, Anna C. Pavlick

**Affiliations:** 1 Department of Medicine, NYU Langone Health, New York, NY USA; 2 Interdisciplinary Melanoma Cooperative Group, NYU Langone Health, New York, NY USA; 3 Ronald O. Perelman Department of Dermatology, NYU Langone Health, New York, NY USA; 4 Department of Surgery, NYU Langone Health, New York, NY USA; 5 Laura and Isaac Perlmutter Cancer Center, NYU Langone Health, 160 East 34th Street, 9N Floor, New York, NY 10016 USA; 6 Department of Medicine, Icahn School of Medicine at Mount Sinai, New York, NY USA; 7 Tisch Cancer Institute, Icahn School of Medicine at Mount Sinai, New York, NY USA; 8 Institute for Health Care Delivery Science, Icahn School of Medicine at Mount Sinai, New York, NY USA; 9 Parker Institute for Cancer Immunotherapy, Extramural Member, New York, NY USA

**Keywords:** Melanoma, NY-ESO-1, Cancer Testis Antigen, Tumor antigen, Vaccine, Immunotherapy, Adjuvant, PD-1, Nivolumab, CTLA-4

## Abstract

**Background:**

Cancer-testis antigen NY-ESO-1 is a highly immunogenic melanoma antigen which has been incorporated into adjuvant vaccine clinical trials. Three such early-phase trials were conducted at our center among patients with high-risk resected melanoma. We herein report on the pooled long-term survival outcomes of these patients in comparison to historical controls.

**Methods:**

All melanoma patients treated at NYU Langone Health under any of three prospective adjuvant NY-ESO-1 vaccine trials were retrospectively pooled into a single cohort. All such patients with stage III melanoma were subsequently compared to historical control patients identified via a prospective institutional database with protocol-driven follow-up. Survival times were calculated using the Kaplan-Meier method, and Cox proportional hazard models were employed to identify significant prognostic factors and control for confounding variables.

**Results:**

A total of 91 patients were treated with an NY-ESO-1 vaccine for the treatment of high-risk resected melanoma. Of this group, 67 patients were stage III and were selected for comparative analysis with 123 historical control patients with resected stage III melanoma who received no adjuvant therapy. Among the pooled vaccine cohort (median follow-up 61 months), the estimated median recurrence-free survival was 45 months, while the median overall survival was not yet reached. In the control cohort of 123 patients (median follow-up 30 months), the estimated median recurrence-free and overall survival were 22 and 58 months, respectively. Within the retrospective stage III cohort, NY-ESO-1 vaccine was associated with decreased risk of recurrence (HR = 0.56, *p* < 0.01) and death (HR = 0.51, *p* = 0.01). Upon controlling for sub-stage, the adjuvant NY-ESO-1 clinical trial cohort continued to exhibit decreased risk of recurrence (HR = 0.45, *p* < 0.01) and death (HR = 0.40, *p* < 0.01).

**Conclusions:**

In this small retrospective cohort of resected stage III melanoma patients, adjuvant NY-ESO-1 vaccine immunotherapy was associated with longer recurrence-free and overall survival relative to historical controls. These data support the continued investigation of adjuvant NY-ESO-1 based immunotherapy regimens in melanoma.

## Background

Despite transformative advances in cancer immunotherapy with respect to checkpoint inhibition – especially in the treatment of melanoma [1–4] – tumor antigen-based vaccine immunotherapy has not consistently been found to generate a substantial anti-neoplastic effect. To this day, sipuleucel-T (Provenge), a cell-based vaccine for the treatment of metastatic castration-resistant prostate cancer [5], remains the only antigen-specific cancer vaccine to have garnered approval from the United States Food and Drug Administration (FDA), and has not been widely-adopted in clinical practice. In contrast, talminogene lapherparevec (TVEC), a genetically-modified herpes simplex virus considered to be an in situ vaccine [6], has been FDA-approved for intratumoral injection of locally-recurrent melanoma, and is more commonly used. Despite evidence of antineoplastic activity, TVEC has not been found to definitively improve survival, [7] and it elicits an immune response by a less direct mechanism than that of tumor-associated antigen-based vaccine immunotherapy.

Since the early 2000s, several tumor-associated antigens [8–10], most notably a class of proteins known as cancer testis antigens (CTAs) [11, 12], have been adopted for vaccine immunotherapy clinical trials, three of which were conducted at our institution in the high-risk melanoma population [13–15]. CTAs are a family of proteins expressed on gametes and trophoblasts as well as various tumor types, but not on normal diploid tissues. Given the immune-privileged nature of human gametes and trophoblasts, CTAs may be therapeutically targeted without substantial risk of immune-mediated off-target effects. Additionally, CTAs are generally recognized and targeted by CD8+ T lymphocytes, making them promising agents for cancer vaccine immunotherapy [16]. In particular, NY-ESO-1, a member of the class of CTAs, is known to induce both humoral [17] and cellular [18] immune responses, and is expressed on a variety of different tumor types [19–26], most notably melanoma [27], synovial sarcoma [28], and ovarian cancer [29].

Although vaccine development has been gradual relative to the exciting progress in immune checkpoint inhibition, several studies have indicated that vaccine-based immunotherapy is capable of inciting a tumor-specific immune response in vivo [11, 30–37] and may be associated with improved survival [9, 38, 39] and tumor regression in the metastatic setting [9, 27, 28]. In fact, renewed interest in NY-ESO-1 directed immunotherapy has given rise to several recent early-phase clinical trials [40] in melanoma as well as in many other cancer types, including both solid tumors and hematologic malignancies. Given the suggestion of possible clinical benefit and scarcity of outcomes data pertaining to vaccine-based immunotherapy, we herein examine the pooled long-term outcomes of three early-phase adjuvant NY-ESO-1 vaccine clinical trials in high-risk resected melanoma.

## Methods

### Adjuvant NY-ESO-1 vaccine cohort

All patients treated for melanoma on any of three prospective phase I and phase II trials (NCT00124124, NCT00821652, and NCT01079741) were retrospectively enrolled in the present study. This retrospective study was approved by the NYU Institutional Review Board (IRB), which granted a waiver of informed consent. Data was collected by retrospective chart review, including: age, sex, race, thickness, ulceration, American Joint Committee on Cancer (AJCC) stage (7th edition staging manual), histologic subtype, time to recurrence, sites of recurrence, additional surgeries, time to last follow-up, and status at last follow-up. Given the preponderance of stage III patients among these clinical trials, and the expectation that a substantial number of survival events had occurred by the time of this analysis among the stage III patients, this cohort was selected for comparative analysis with historical controls.

#### NCT00124124: Comparison of dendritic cells versus montanide as adjuvants in a melanoma vaccine [13]

This phase I trial enrolled adult patients with stage IIB, IIC, or III surgically-resected melanoma between 2005 and 2008. Patients were randomly assigned to receive either: HLA-A0201 restricted melanoma-associated peptides (including NY-ESO-1 and Melan A), keyhole limpet haemocyanin (immunogenic vaccine antigen), and either: peptide-loaded dendritic cells or montanide (immunogenic vaccine adjuvant; SEPPIC, Paris, France). Because these patients received peptides as opposed to whole protein, they were required to be HLA-A2 positive by genotype in order to be eligible for the study.

#### NCT00821652: Randomized, double blind, placebo-controlled topical resiquimod adjuvant for NY-ESO-1 protein vaccination [11, 14]

This dose-finding and expansion phase I trial enrolled – between 2009 and 2010 – the same patient population as NCT00124124, with the addition of resected stage IV patients. The dose-finding phase of the study treated patients with progressive doses of topical resiquimod (toll-like receptor agonist) in addition to NY-ESO-1 whole protein and montanide. In the expansion part of the study, patients were randomized to receive NY-ESO-1 whole protein and montanide with topical resiquimod or placebo.

#### NCT01079741: Safety study of adjuvant vaccine to treat melanoma patients [15, 41]

This phase I/II dose escalation and expansion trial enrolled the same patient population as NCT00821652, including resected stage IIB-IV melanoma, between 2010 and 2013. Patients in the dose expansion phase were treated with NY-ESO-1 whole protein, montanide, and escalating doses of poly-ICLC (immunogenic vaccine adjuvant). In the dose expansion phase, patients received NY-ESO-1 whole protein and poly-ICLC with or without montanide.

### Historical control cohort

All consenting melanoma patients who present to NYU Langone Health for diagnosis and/or treatment of melanoma are enrolled in the NYU Interdisciplinary Melanoma Cooperative Group database and biorepository, which enables collection of a comprehensive set of demographic, clinical, and pathologic data from each consenting patient, including: age, sex, race, thickness, ulceration, AJCC stage, histologic subtype, time to recurrence, pattern of recurrence, time to last follow-up, and melanoma status at last follow-up. These data are regularly updated via a protocol-driven follow-up schedule. This protocol has been approved by the NYU IRB, and all patients provide informed consent at the time of enrollment. All patients in the database who underwent surgical resection for stage III melanoma and received no systemic adjuvant therapy were included for analysis.

### Statistical methods

Descriptive statistics were performed on both the entire NY-ESO-1 vaccine cohort – including patients of all stages – as well as the historical control cohort. Continuous variables (e.g. age and thickness) were analyzed using Student’s t test; thickness was log-transformed due to its non-normal distribution. Categorical variables (e.g. ulceration and stage) were analyzed using Fisher’s exact test or the chi-squared test, where appropriate. The Kaplan-Meier method was utilized to test for differences in recurrence-free and overall survival of stage III patients between the three adjuvant clinical trials. The Kaplan-Meier method was also used to test for differences between the pooled adjuvant NY-ESO-1 vaccine cohort and the control cohort. Univariate and multivariate Cox proportional hazard regression models were performed to examine the impact of known melanoma prognostic factors as well as adjuvant NY-ESO-1 vaccine on post-surgical recurrence and death. For stage III patients with recurrent melanoma, Fisher’s exact test was used to examine differences in recurrence pattern (resectable versus non-resectable) between the vaccine and control cohorts.

Of note, although T cell response data exists for these three trials, the complete dataset has not yet been compiled, as the immunologic data analysis for NCT01079741 is incomplete and will likely be reported separately in a future manuscript once all analyses are complete.

## Results

### Clinical trial patient characteristics

A total of 91 melanoma patients received an adjuvant NY-ESO-1 vaccine on one of the three clinical trials (Table 1). Owing to differences in inclusion criteria among the three trials, there was a significant difference in the composition of patients with respect to AJCC stage (*p* = 0.01), associated with the enrollment of resected stage IV patients on NCT00821652 and NCT01079741. Otherwise, across these three trials, there were no other significant differences in baseline age, sex, ulceration, histologic subtype, and anatomic site. While NCT00821652 and NCT01079741 were comprised mostly of men, the NCT00124124 cohort was female predominant, though this trend did not reach statistical significance (*p* = 0.24). Of note, owing to the post-hoc nature of this analysis and an interval change in medical record system, there were many missing data fields among this cohort, especially with respect to thickness, ulceration, and histologic subtype.

**Table 1:** Baseline patient characteristics among all three adjuvant NY-ESO-1 clinical trials

	NCT00821652	NCT01079741	NCT00124124	
	*N* = 22	*N* = 35	*N* = 34	
	N(%)/Mean(SD)	N(%)/Mean(SD)	N(%)/Mean(SD)	*P*-Value
Age	59.68 (13.60)	55.49 (15.16)	56.38 (11.82)	0.5134
Sex				0.2446
Female	9 (40.91)	14 (40.00)	20 (58.82)	
Male	13 (59.09)	21 (60.00)	14 (41.18)	
Thickness^a^	2.63 (1.64)	2.22 (1.84)	4.48 (5.63)	0.0122
Ulceration				0.1916
Present	7 (31.82)	11 (31.43)	18 (52.94)	
Absent	10 (45.45)	17 (48.57)	14 (41.18)	
Undetermined	5 (22.73)	7 (20.00)	2 (5.88)	
AJCC Stage				0.0146
IIB	0 (0.00)	3 (8.57)	7 (20.59)	
IIC	1 (4.55)	1 (2.86)	0 (0.00)	
IIIA	5 (22.73)	4 (11.43)	6 (17.65)	
IIIB	5 (22.73)	11 (31.43)	10 (29.41)	
IIIC	4 (18.18)	12 (34.29)	10 (29.41)	
IV	7 (31.82)	4 (11.43)	0 (0.00)	
Undetermined	0 (0.00)	0 (0.00)	1 (2.94)	
Histologic Subtype				0.5588
Nodular	6 (27.27)	7 (20.00)	12 (35.29)	
Superficial Spreading	4 (18.18)	5 (14.29)	7 (20.59)	
Other	3 (13.64)	2 (5.71)	4 (11.76)	
Undetermined	9 (40.91)	21 (60.00)	11 (32.35)	
Primary site				0.2624
Anterior Trunk	0 (0.00)	4 (11.43)	6 (17.65)	
Arms	3 (13.64)	5 (14.29)	1 (2.94)	
Head/Neck	5 (22.73)	3 (8.57)	6 (17.65)	
Legs	8 (36.36)	15 (42.86)	12 (35.29)	
Posterior Trunk	3 (13.64)	6 (17.14)	8 (23.53)	
Unknown	3 (13.64)	2 (5.71)	1 (2.94)	

^a^missing values for thickness: NCT00821652: 4; NCT01079741: 5;NCT00124124: 1

**Retrospective cohort patient characteristics.**

**Fig. 1:**
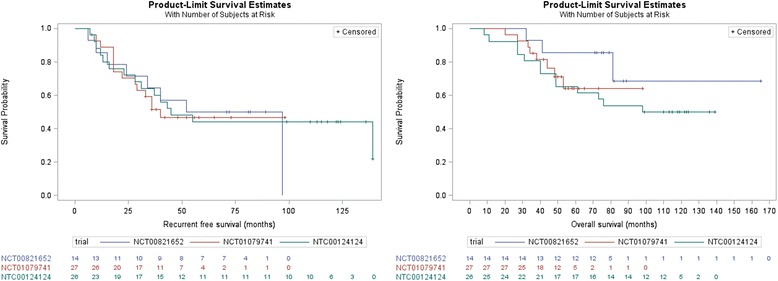
Recurrence-free (left, log-rank *p* = 0.98) and overall survival (right, log-rank *p* = 0.37) of all adjuvant stage III NY-ESO-1 vaccine clinical trial patients stratified by each of the three individual trials

As previously described, a total of 67 stage III clinical trial patients were selected for comparison with an historical control cohort of 123 stage III patients (Table 2). As a whole, the vaccine patients were all enrolled between 2001 and 2012 (interquartile range 2006–2010), before the widespread use of immunotherapy and targeted therapy, though it must be acknowledged that both ipilimumab as well as *BRAF* inhibitors were both widely used for the treatment of metastatic melanoma after 2011. The historical control group was enrolled between 1986 and 2014 (interquartile range 2007–2014), and no patients in either cohort received adjuvant checkpoint inhibitor or adjuvant *BRAF* targeted therapy in any form. Among the stage III patients treated with an adjuvant NY-ESO-1 vaccine, there were no statistically significant differences in recurrence-free or overall survival between the clinical trials (Fig. 1), further supporting the analysis of these patients in a pooled fashion. There were no significant differences between the stage III vaccine patients and the stage III control patients with respect to age, though we did observe a trend toward younger patients among the treatment cohort. We did find a significant difference with respect to the stage III sub-stage between the two patient cohorts (*p* < 0.01), with vaccine patients diagnosed more frequently with stage IIIC (39% vs 20%) and less frequently with IIIA (22% vs 39%). Of note, both patient cohorts exhibit a male-predominance, though the male-female distribution is not different between the vaccine and control groups. Missing data with respect to thickness, ulceration, and histologic subtype hinders comparison of these parameters across cohorts.

**Table 2:** Baseline patient characteristics among stage III patients comprising the retrospective cohort

	Vaccine	No Adjuvant	
	*N* = 67	*N* = 123	
	N(%)/Mean(SD)	N(%)/Mean(SD)	*P*-Value
Age	56.00 (13.44)	60.05 (16.44)	0.086
Sex			0.2854
Female	31 (46.27)	47 (38.21)	
Male	36 (53.73)	76 (61.79)	
Thickness^a^	3.12 (4.43)	4.18 (4.46)	0.009
Ulceration			0.3511
Present	23 (34.33)	52 (42.28)	
Absent	32 (47.76)	57 (46.34)	
Undetermined	12 (17.91)	14 (11.38)	
AJCC Stage			0.0082
IIIA	15 (22.39)	48 (39.02)	
IIIB	26 (38.81)	51 (41.46)	
IIIC	26 (38.81)	24 (19.51)	
Histologic Subtype			0.0435
Nodular	17 (25.37)	52 (42.28)	
Superficial Spreading	12 (17.91)	26 (21.14)	
Other	6 (8.96)	13 (10.57)	
Undetermined	32 (47.76)	32 (26.02)	
Other Adjuvant Treatment			–
GM-CSF	6 (8.96)	Not Applicable	
Interferon	6 (8.96)		
Isolated Limb Infusion	3 (4.48)		
Radiation	22 (32.84)		

^a^missing values for thickness: vaccine: 10; no adjuvant: 13

^a^thickness not normally-distributed, log-transformed for statistical test

### Adjuvant NY-ESO-1 vaccine is associated with prolonged survival

**Fig. 2:**
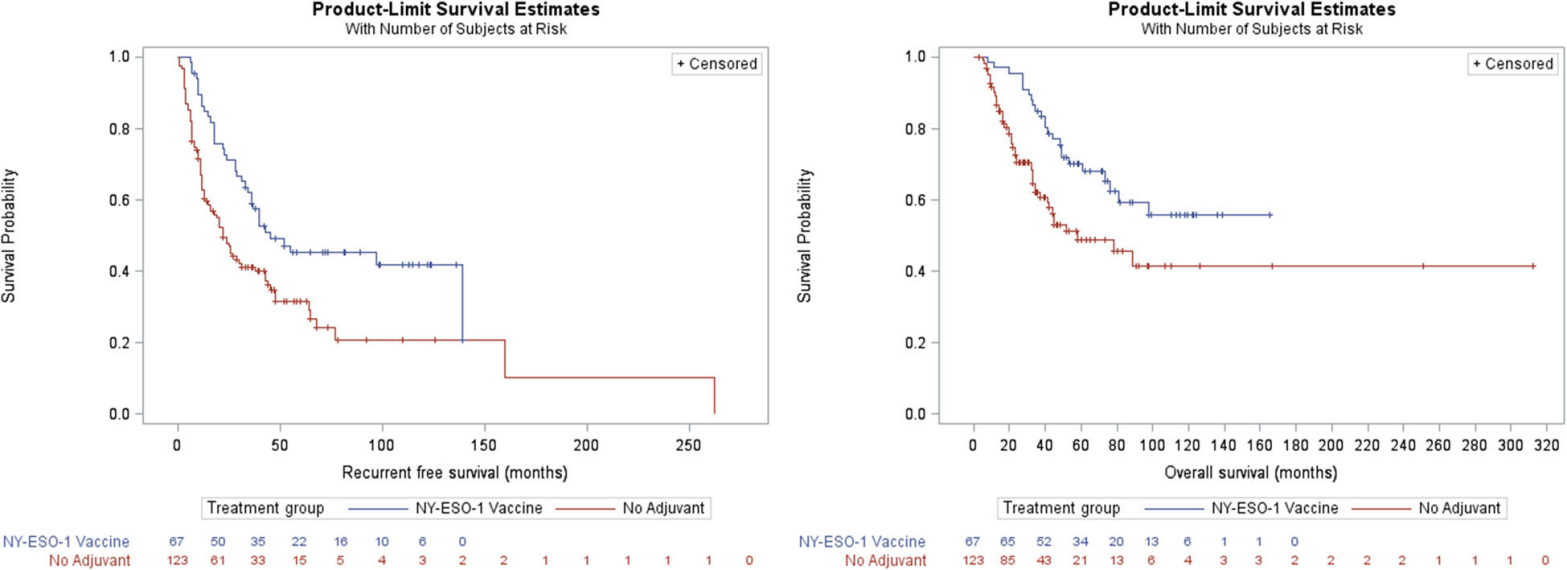
Recurrence-free (left, *p* < 0.01) and overall survival (right, *p* = 0.01) among all stage III patients stratified by adjuvant NY-ESO-1 vaccine versus no adjuvant therapy

Among the 67 stage III vaccine cohort, at a median follow-up time of 61 months, 37 patients had recurred (55%) and 24 had died (36%). In comparison, the control cohort of 123 patients was found to have a shorter median follow-up time of just 30 months, during which 82 patients recurred (67%) and 50 died (41%). Despite the longer follow-up among the adjuvant vaccine group, the median overall survival was not reached in this cohort (Fig. 2). Univariate hazard analysis (Table 3) recapitulated the expected impact of known melanoma prognostic variables such as stage III sub-stage (IIIC vs. IIIA HR = 1.46), thickness (HR = 1.10), ulceration (HR = 2.19), and age (HR = 1.03). In addition, NY-ESO-1 vaccine was associated with significantly decreased risk of recurrence (HR = 0.56, *p* < 0.01) and death (HR = 0.51, *p* = 0.01) within this retrospective cohort of resected stage III patients. Among the retrospective stage III cohort, adjuvant NY-ESO-1 vaccine was associated with a prolonged estimated median recurrence-free survival of 45 months relative to 22 months in the no adjuvant cohort (log-rank *p* < 0.01, Fig. 2), as well as a prolonged estimated median overall survival which was not reached relative to 58 months in the control cohort (log-rank *p* = 0.01, Fig. 2). Given the difference in sub-stage distribution between the vaccine and historical control cohorts, as well as the expectation that this factor would be the dominant prognosticator among patients with resected stage III melanoma, a multivariate Cox model was constructed utilizing both AJCC stage III sub-stage as well as NY-ESO-1 vaccine versus no adjuvant therapy. Controlling for the effect of stage III sub-stage on recurrence and survival, the multivariate model (Table 3) continued to demonstrate a marked reduction in the risk of recurrence (HR = 0.45, *p* < 0.01) and death (HR = 0.40, *p* < 0.01) associated with adjuvant NY-ESO-1 vaccine.

**Table 3:** Cox proportional hazard models of recurrence-free and overall survival among the retrospective stage III cohort

		Univariate Hazard Model					
		Recurrence-Free Survival			Overall Survival		
		HR	95% CI	*P*-value	HR	95% CI	*P*-value
NY-ESO-1 Vaccine		0.555	(0.374, 0.822)	0.0034	0.507	(0.310, 0.831)	0.007
Thickness (1 mm)		1.079	(1.044, 1.116)	< 0.0001	1.101	(1.062, 1.141)	< 0.0001
Ulceration	Absent	1			1		
	Present	2.206	(1.490, 3.266)	< 0.0001	2.185	(1.336, 3.573)	0.0019
	Undetermined	1.079	(0.600, 1.940)	0.8002	0.953	(0.413, 2.196)	0.9093
Age (1 year)		1.015	(1.002, 1.027)	0.0201	1.025	(1.008, 1.042)	0.0033
AJCC Stage	IIIA	1			1		
	IIIB	1.141	(0.730, 1.783)	0.5627	1.005	(0.643, 1.570)	0.9842
	IIIC	1.795	(1.130, 2.850)	0.0132	1.457	(0.915, 2.319)	0.1129
		Multivariate Hazard Model					
		Recurrence-Free Survival			Overall Survival		
		HR	95% CI	*P*-value	HR	95% CI	*P*-value
NY-ESO-1 Vaccine		0.454	(0.301, 0.685)	0.0002	0.403	(0.269, 0.604)	< 0.0001
AJCC Stage	IIIA	1	(0.561, 1.370)		1		
	IIIB	1.225	(0.783, 1.916)	0.3744	1.028	(0.658, 1.608)	0.9026
	IIIC	2.348	(1.452, 3.797)	0.0005	1.792	(1.114, 2.883)	0.0161

### Adjuvant NY-ESO-1 vaccine is not associated with a significantly different pattern of recurrence

**Fig. 3:**
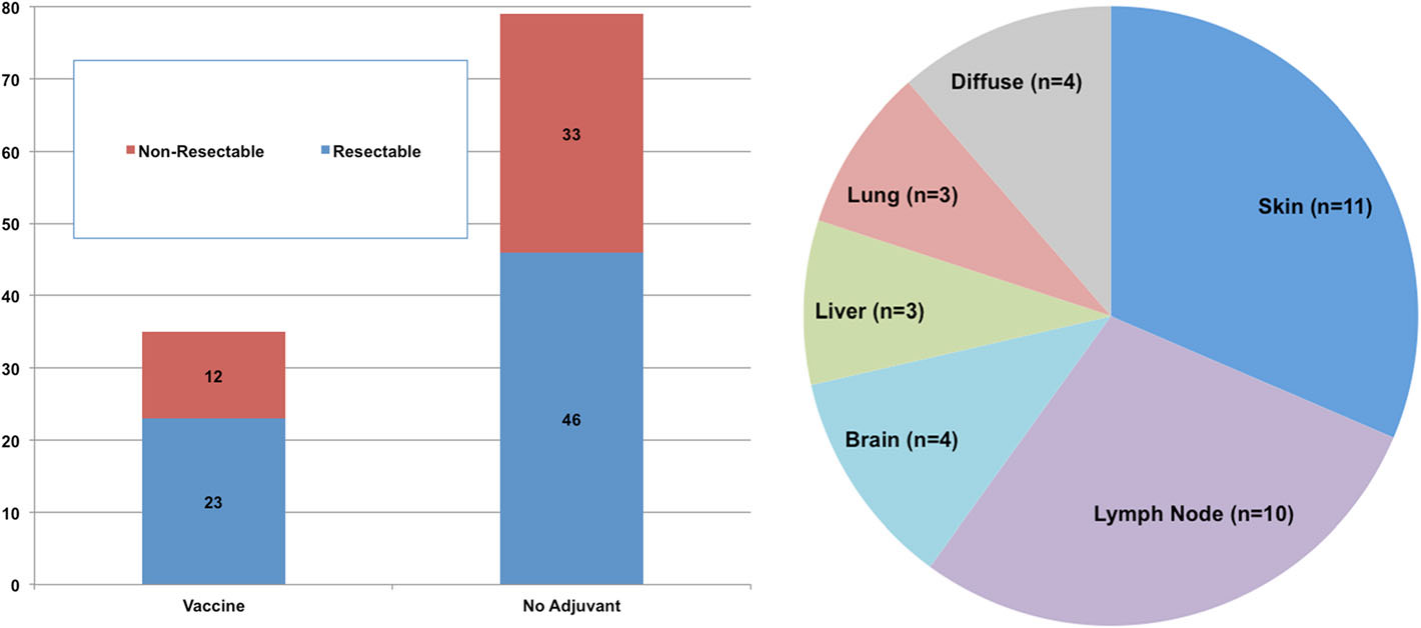
Recurrence patterns among retrospective stage III cohort stratified by vaccine versus no adjuvant therapy (left, *p* = 0.5) indicating the number of patients with resectable versus non-resectable recurrences, and specific sites of disease recurrence among the stage III adjuvant NY-ESO1 vaccine cohort (right, *n* = 35)

Among the pooled vaccine cohort of 67, a total of 35 patients recurred post-vaccine; in comparison, 82 patients among the historical control cohort had recurred at last follow-up. Of the 35 NY-ESO-1 vaccine patients who recurred, 23 (66%) were surgically resectable at the time of recurrence, compared to 46 (58%) in the control cohort (Fig. 3). Although we observed a modestly increased prevalence of resectability at the time of disease recurrence among adjuvant vaccine patients, this trend did not reach statistical significance. The most common anatomic patterns of recurrence among the vaccine cohort were: cutaneous (*n* = 11), lymph node (*n* = 10), and brain (*n* = 4), and just 4 patients recurred with diffuse metastatic disease involving multiple organ systems.

## Discussion

In this retrospective analysis, we have demonstrated that patients treated at our institution on early phase clinical trials of adjuvant NY-ESO-1-based vaccine immunotherapy exhibited very good long-term survival outcomes in resected stage III melanoma. Specifically, in comparison to our single-institution historical control cohort, patients who were treated on an adjuvant NY-ESO-1 vaccine trial experienced significantly longer recurrence-free and overall survival. With a treatment cohort of 67 patients and a median follow-up time of over 5 years, the present study likely represents the most robust long-term survival analyses of adjuvant NY-ESO-1 vaccines to date.

The comprehensive body of evidence for an NY-ESO-1-mediated humoral and cellular immune response to cancer provides a strong rationale for NY-ESO-1-based immunotherapy. In fact, the observation that NY-ESO-1 induces both humoral and cellular immunity [18] lead to its ultimate development for the purposes of anticancer vaccines and cell-based immunotherapeutics. Several phase I clinical trials [11, 29, 31, 32, 35, 36, 42, 43] – mostly conducted in the melanoma population – have demonstrated the ability to utilize NY-ESO-1 peptide and whole protein to induce both NY-ESO-specific antibody and T lymphocyte responses in vivo. While most of these trials utilized specific NY-ESO-1 peptides which have been demonstrated to elicit in vivo immune responses in an HLA-A2-restricted fashion, others [11, 36, 42] used NY-ESO-1 whole protein. Of note, the 3 early-phase adjuvant NY-ESO-1 trials analyzed in the present study utilized both a peptide-based vaccine [13] as well as whole protein regimens [14, 15]. Regardless of the specific NY-ESO-1 vaccine regimen, available data supports the notion that intracutaneous NY-ESO-1 (either whole protein or peptides) vaccination is capable of NY-ESO-1 seroconversion [29, 36] as well as induction of measurable NY-ESO-1-specific CD4+ [11, 35] and CD8+ [29, 31, 32, 36, 43] T lymphocyte populations. Given the similarity in survival outcomes across all three of these trials and the absence of a clear signal in published data supporting either peptide or whole protein, we feel it is reasonable to consider the patients treated on these trials as a single cohort. Relative to the strong evidence to suggest that the immunogenicity of NY-ESO-1 may be inducible by vaccination, there exists a paucity of outcomes data regarding the clinical efficacy of NY-ESO-1-based vaccines.

A limited cadre of studies has, however, sought to correlate the inducible immunity against NY-ESO-1 with clinical outcomes of patients. In a small non-randomized clinical trial in advanced solid tumors (mostly metastatic melanoma) conducted by Jaeger, et al. [30], peptide vaccination was associated with induction of an NY-ESO-1-specific CD8+ T cell expansion. Of five NY-ESO-1 seropositive patients, three exhibited disease stabilization, and a single patient exhibited seroconversion with respect to NY-ESO-1 antibodies. Another small early-phase trial of NY-ESO-1 peptide in advanced solid tumors, mostly comprised of non-resectable melanoma, was conducted by Karbach, et al. [44]. Interestingly, this study reported that among the nine patients who developed a measurable CD8+ T cell response to vaccination, six were still alive after 2 years of follow-up. More recently, Odunsi, et al. [45] have reported on the efficacy of a recombinant viral vector expressing NY-ESO-1 in two phase II clinical trials conducted in epithelial ovarian cancer and stage III and IV melanoma. Among 25 melanoma patients, two objective responses (one complete response and one partial response) were observed, and the authors reported an impressive 72% disease control rate. Additionally, this study found a 9 month median progression-free survival as well as a 48 month median overall survival, which are particularly impressive given the predominance of stage IV patients among this cohort. Furthermore, as Diem et al. [46] have noted, the majority of the clinical benefit associated with immunotherapy is likely derived among patients with a low burden of disease, and it could be inferred that immunotherapy, including tumor-associated antigen vaccines, exerts maximal influence on the course of disease when utilized in the adjuvant setting where there is minimal residual disease following surgical resection.

Longer follow-up data from an adjuvant placebo-controlled trial conducted by Davis, et al. [33] also suggests clinical benefit associated with adjuvant NY-ESO-1 vaccination. In this randomized placebo-controlled study of adjuvant recombinant NY-ESO-1 whole protein, a total of 42 high-risk resected melanoma patients were enrolled. Interestingly, every patient who was treated with both recombinant NY-ESO-1 plus ISCOMATRIX developed humoral immunity, and at a median follow-up of just over 2 years, a disproportionate number of relapses had occurred among the placebo cohort. While five of seven placebo patients had relapsed, only two of 19 patients treated with NY-ESO-1 with ISCOMATRIX adjuvant had relapsed. At a median follow-up of 1430 days, Nicholaou, et al. [38] published an updated analysis of this cohort demonstrating similar findings, with relapses among only five of 19 patients in the full treatment cohort compared to six of seven patients in the placebo cohort. Of these relapsed patients, a majority of the patients in the treatment cohort exhibited persisting humoral and cellular immunity relative to zero patients in the placebo cohort, suggesting that induced immunity toward NY-ESO-1 is perhaps mediating the delay in melanoma recurrence. Although these studies yield highly suggestive evidence of the efficacy of NY-ESO-1 based vaccine immunotherapy, no overall survival analysis was performed, and no systematic analysis was performed to control for disease stage despite the fact that enrolled melanoma patients ranged from stage Ib to resected stage IV.

Several authors [47–49] have reported data to support the notion that the efficacy of vaccine immunotherapy is perhaps associated with specific HLA genotypes. Of particular interest is work by Carson, et al. [47] who describe the long-term follow-up of an adjuvant melanoma cell lysate-based vaccine in the treatment of resected stage II melanoma. Specifically, the authors found an association between HLA-A2 and improved recurrence-free and overall survival. This finding is of interest given the HLA-A2 restriction among patients treated with adjuvant NY-ESO-1 vaccine in the two peptide-based trials included in the present study. However, the lysate-based vaccine trial reported by Carson et al. did not include NY-ESO-1 [49]. Furthermore, HLA typing is not available for the NYU historical control patients, which precludes a comparative analysis of the NYU cohort on the basis of HLA genotype.

In addition to recurrence-free and overall survival analyses, we have also analyzed our institutional cohort with respect to the pattern of disease recurrence. This examination was motivated by the qualitative observation that some patients enrolled on these early phase vaccine clinical trials seemed to exhibit a more limited pattern of disease recurrence such that they were able to undergo multiple surgical resections rendering them free of disease. This idea was supported by the observation made by Jager, et al. [50], who reported on the immunologic and survival outcomes of an early phase recombinant viral-NY-ESO-1 vaccine study in advanced solid tumors. Of interest was a single patient with multiply recurrent stage III melanoma who developed an additional isolated nodal metastasis while on-treatment. The patient underwent surgical resection of the involved node and continued to receive vaccine; this patient subsequently remained free of disease for over 5 years. Although there is a slight trend toward surgical resectability among our vaccine cohort, this tendency did not reach the level of statistical significance. Larger prospective studies would be required to determine what effect, if any, NY-ESO-1 vaccines exert on the pattern of melanoma recurrence.

The present study has several important limitations. Firstly, these analyses result from a post-hoc analysis of retrospective data which is complicated further by the significant treatment heterogeneity with respect to active vaccine antigens (peptide versus whole protein, NY-ESO-1 alone versus a combination of peptides), vaccine adjuvants (e.g. montanide, poly-ICLC, etc.), and vaccination mechanism (matured dendritic cells versus direct antigen injection). Secondly, we have relied on the use of historical control patients, who received no adjuvant therapy. While the clinical trial patients were necessarily with good performance status and no evidence of any imminent medical co-morbidities, the historical control cohort comprised patients who received no adjuvant therapy, which could reflect high-risk baseline characteristics not captured in this analysis. Despite the limitations of using historical controls, the survival data of the NYU control group is quite comparable to the control arm of EORTC 18071, an adjuvant trial of ipilimumab in high-risk resected melanoma, with 3-year overall survival rates of approximately 65 and 60% in EORTC and NYU, respectively. In addition, the difference in median follow-up times between the vaccine group and the historical controls is a potential source of bias in this analysis; however, this difference is predominantly driven by both ongoing recruitment of newer NYU melanoma registry patients, for whom less follow-up is available, and the observed shorter recurrence-free and overall survival among the control patients relative to patients receiving adjuvant NY-ESO-1. Lastly, the widespread use of immune checkpoint inhibitors and *BRAF* targeted therapy revolutionized the treatment of metastatic melanoma in the years following closure of these trials. Although the patients enrolled in these trials were accrued during roughly the same time period as the historical controls were diagnosed at our institution, it is very likely that there exists heterogeneity in the post-recurrence treatments these patients later received.

The adjuvant treatment landscape in high-risk resected melanoma is actively evolving, with increasing emphasis on immune checkpoint inhibition [2, 4] as well as *BRAF* targeted therapy [51]. Ipilimumab, the anti- cytotoxic T lymphocyte antigen 4 (anti-CTLA-4) antibody, was FDA-approved in 2015 for the adjuvant treatment of resected stage III melanoma, though it is infrequently used in the adjuvant setting due to its unfavorable side effect profile [4]. More importantly, the recent study conducted by Weber, et al. [2] demonstrated significant efficacy of the anti- programmed death receptor 1 (anti-PD-1) antibody, nivolumab, in preventing melanoma recurrence when administered following complete surgical resection. Of note, this study found a moderately low rate of immune-related adverse events associated with nivolumab relative to ipilimumab, making anti-PD-1 immunotherapy an attractive choice in the adjuvant setting. However, nivolumab was only recently been FDA-approved for the adjuvant treatment of melanoma in late 2017, and post-marketing experience in the adjuvant setting is limited. Notably, NY-ESO-1 vaccine immunotherapy has also been found to be remarkably well-tolerated [11, 41]. Although anti-PD-1 checkpoint inhibitor immunotherapy will almost certainly form the backbone of adjuvant regimens in melanoma, this study supports a possible role for the investigative addition of adjuvant NY-ESO-1 vaccine immunotherapy in the setting of prospective clinical trials.

## Conclusions

In this small retrospective cohort of resected stage III melanoma, adjuvant NY-ESO-1 based vaccine regimens appear to be associated with improved recurrence-free and overall survival relative to historical controls. In conjunction with the well-established body of literature supporting the immunogenicity of NY-ESO-1, these results support the continued investigation of adjuvant NY-ESO-1 vaccine immunotherapy. Further study is needed to prospectively validate the reported clinical benefit and determine the optimal vaccine regimen, especially in combination with well-established immune checkpoint inhibitors.

## Abbreviations

AJCC: American Joint Committee on Cancer
CTA: cancer testis antigens
CTLA-4: cytotoxic T lymphocyte antigen 4
FDA: Food and Drug Administration
IRB: Institutional Review Board
PD-1: programmed death receptor 1
TVEC: talminogene lapherparevec
